# Breast Cancer Vaccine Containing a Novel Toll-like Receptor 7 Agonist and an Aluminum Adjuvant Exerts Antitumor Effects

**DOI:** 10.3390/ijms232315130

**Published:** 2022-12-01

**Authors:** Shuquan Zhang, Yu Liu, Ji Zhou, Jiaxin Wang, Guangyi Jin, Xiaodong Wang

**Affiliations:** School of Pharmaceutical Sciences, Nation-Regional Engineering Lab for Synthetic Biology of Medicine, International Cancer Center, Shenzhen University Health Science Center, Shenzhen 518060, China

**Keywords:** TLR7 (Toll-like receptor 7), MUC1 (Mucin 1), aluminum adjuvant, tumor vaccine, immunotherapy

## Abstract

Mucin 1 (MUC1) has received increasing attention due to its high expression in breast cancer, in which MUC1 acts as a cancer antigen. Our group has been committed to the development of small-molecule TLR7 (Toll-like receptor 7) agonists, which have been widely investigated in the field of tumor immunotherapy. In the present study, we constructed a novel tumor vaccine (SZU251 + MUC1 + Al) containing MUC1 and two types of adjuvants: a TLR7 agonist (SZU251) and an aluminum adjuvant (Al). Immunostimulatory responses were first verified in vitro, where the vaccine promoted the release of cytokines and the expression of costimulatory molecules in mouse BMDCs (bone marrow dendritic cells) and spleen lymphocytes. Then, we demonstrated that SZU251 + MUC1 + Al was effective and safe against a tumor expressing the MUC1 antigen in both prophylactic and therapeutic schedules in vivo. The immune responses in vivo were attributed to the increase in specific humoral and cellular immunity, including antibody titers, CD4^+^, CD8^+^ and activated CD8^+^ T cells. Therefore, our vaccine candidate may have beneficial effects in the prevention and treatment of breast cancer patients.

## 1. Introduction

Toll-like receptors (TLRs) are a family of integral membrane proteins, primarily localized in immune cells such as dendritic cells (DCs) and macrophages. TLRs, recognizing pathogen-associated molecular patterns (PAMPs) in a variety of viruses and microorganisms, are the bridge between innate and adaptive immunity [1,2]. Among all of the TLRs, only TLR7/8 have small-molecule ligands, which are easier to obtain and modify than other biomacromolecules such as TLR4 and TLR9 ligands [3]. Recently, small-molecule TLR7 agonists have been widely investigated in the field of tumor immunotherapy through inducing tumor-specific immune responses and reducing the tumor growth [4,5,6]. Our group has been committed to the development of TLR7 agonists, and SZU101 is one of the representative compounds. We have previously proved that SZU101 enhances the therapeutic efficacy of doxorubicin in a mouse lymphoma model via the generation of systemic immune responses. SZU101 has been used in the form of chemical conjugation with targeted anticancer drugs, including JQ1 (a BET inhibitor) and ibrutinib (a BTK inhibitor) [7]. Furthermore, SZU101 is a successful adjuvant in the development of tumor vaccines, having been conjugated with multiple tumor-associated antigens [8].

Mucin 1 (MUC1) is a membrane-associated glycoprotein overexpressed in many different kinds of epithelial tumor tissues, making it an attractive target for tumor immunotherapy [9]. MUC1 contains a variable number of tandem repeat (VNTR) sequence of twenty amino acids, and five potential O-glycosylation sites are located on the threonine and serine residues of each repeat [10,11]. The peptides from the VNTR sequence are often used as the antigens for tumor vaccines targeting MUC1 [12]. A tumor vaccine has been constructed by covalent attachment of an MUC1 glycopeptide and a T-helper epitope, inducing both humoral and cellular immune responses [13]. Our group developed a novel TLR7 agonist-conjugated MUC1 peptide vaccine that elicited effective immune responses and robust antitumor effects in a mouse breast cancer model [14]. However, appropriate immune stimulators still need to be studied due to the weak immunogenicity of MUC1 peptides.

In this study, we construct a novel tumor vaccine (SZU251 + MUC1 + Al) with three components: a small-molecule TLR7 agonist synthesized for the first time (SZU251), an MUC1-related peptide (MUC1), and an aluminum adjuvant (Al). It is widely accepted that the glycosylated MUC1 peptide is more immunogenic than the non-glycosylated one [13]. However, we already demonstrated the immunogenicity of the non-glycosylated MUC1 peptide in previous work, and the combination of the naked MUC1 sequence and the TLR7 agonist (T7 + MUC1) could be an effective tumor vaccine [14]. Therefore, we have chosen the same naked sequence here in order to verify the improvement of this three-component vaccine (T7 + MUC1 + Al) compared to the previous two-component vaccine (T7 + MUC1). Immunostimulatory effects of the vaccine are first determined in vitro by the detection of cytokines and costimulatory molecules. Then, the humoral and cellular immune responses are verified in a mouse breast cancer model in both prophylactic and therapeutic vaccination schedules. Finally, the vaccine is proved to be well tolerated in the mice by HE staining (hematoxylin–eosin staining) of the major organs and blood tests.

## 2. Results

### 2.1. Chemical Synthesis of the Vaccine Candidates

**Figure 1:**
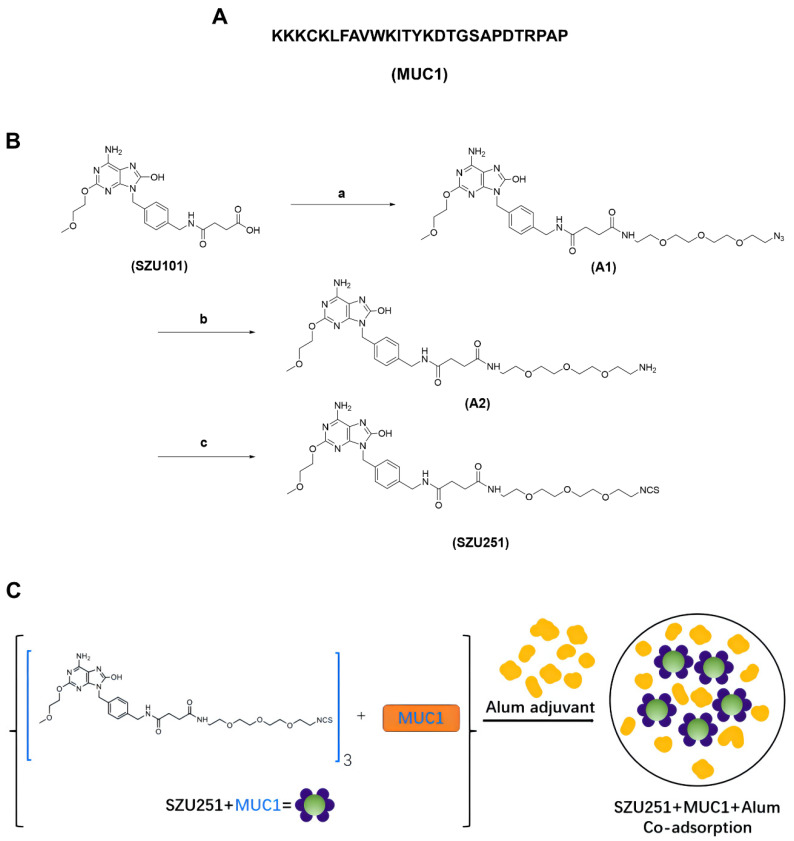
(**A**) Sequence of T-helper linked to MUC1. (**B**) Synthetic route of SZU251. Reagents and conditions: (a) 2-(2-(2-(2-azidoethoxy)ethoxy)ethoxy)ethan-1-amine, HOBT, EDC, DIPEA, DMF, Rt, overnight, LC-MS monitoring reaction, HPLC-purified; (b) Pd/C (10%), MeOH, H_2_, Rt, 2 h, LC-MS monitoring reaction, MeOH spin-dried; (c) (1) CS_2_, TEA, DMF, Rt, overnight; (2) TsCl, Rt, 5 h, LC-MS monitoring reaction, HPLC-purified. (**C**) Scheme of SZU251 + MUC1 + Al.

MUC1 peptide was synthesized by the solid phase method, containing a well-documented MUC1-derived epitope (SAPDTRPAP) and a murine MHC class II restricted T-helper epitope (KLFAVWKITYKDT) (Figure 1A). SZU251 was synthesized for the first time by our group according to the methods shown in Figure 1B. The above-mentioned compounds were confirmed by NMR spectrometry, mass spectrometry or high-performance liquid chromatography (Figures S1 and S2). SZU251 + MUC1 was a mixed formulation at a 3:1 ratio of SZU251 and MUC1, and the 3:1 ratio was selected according to our previous study [14]. All of the vaccine candidates, especially for the in vivo experiments, were mixed with the aluminum adjuvant (Figure 1C). Moreover, the ability of SZU251 for TLR activation was determined on HEK-Blue hTLR7 and hTLR8 cells. SZU251 and R848 (TLR7/8 agonists) induced much more potent TLR7 activation than imiquimod (a TLR7 agonist), while SZU251 and imiquimod could not induce TLR8 activation (Figure S3). Therefore, SZU251 is a novel TLR7 agonist with specificity and selectivity.

### 2.2. In Vitro Immunostimulatory Responses of the Vaccine Candidates

**Figure 2:**
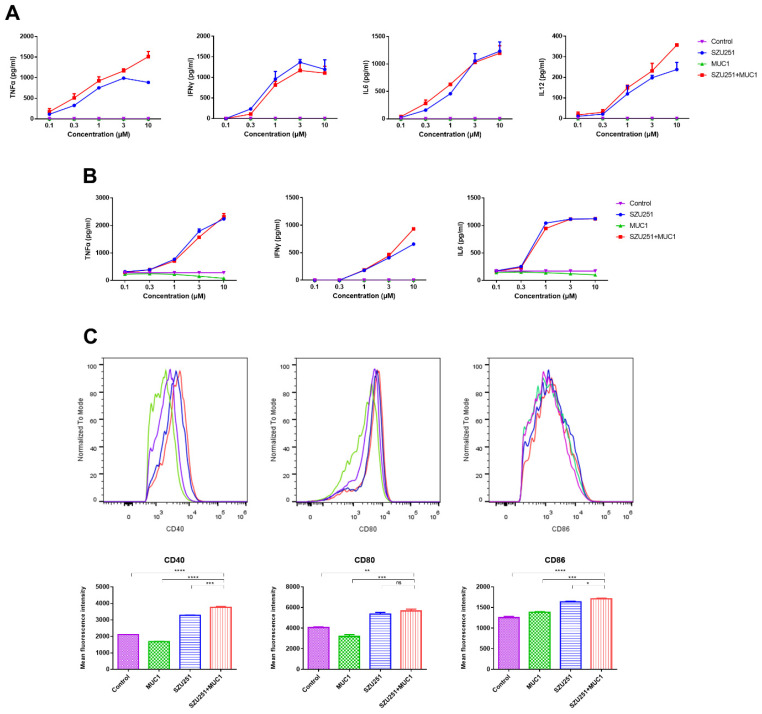
In vitro immunostimulatory responses of the vaccine candidates. (**A**) Induction of TNFα, IFNγ, IL6 and IL12 in mouse spleen lymphocytes after 24 h incubation. (**B**) Induction of TNFα, IFNγ and IL6 in mouse BMDCs after 24 h incubation. (**C**) Maturation of BMDCs was evaluated by measuring the expression of the surface molecules CD40, CD80 and CD86 by flow cytometry after 24 h treatment with 3 µM vaccine candidates. * *p* < 0.05, ** *p* < 0.01, *** *p* < 0.001, **** p < 0.0001, “ns” not significant. TNF, tumor necrosis factor; IFN, interferon; IL, interleukin. Data are presented as the mean ± SE; *n* = 3.

To access the immunostimulatory activity of the vaccine candidates, the release of cytokines was first decided by the ELISA method in mouse spleen lymphocytes and BMDCs (bone marrow dendritic cells). As shown in Figure 2A,B, MUC1 rarely stimulated cytokine production due to its poor immunogenicity. SZU251 and SZU251 + MUC1 displayed similar and remarkable trends of increase in the relevant cytokines in a dose-dependent manner (TNFα, IFNγ, IL6 and IL12 in spleen lymphocytes; TNFα, IFNγ and IL6 in BMDCs). IL12 in BMDCs was also detected but is not displayed here owing to the low secretion levels. Next, maturation of BMDCs was accessed by flow cytometry by detecting the upregulated expression of CD40, CD80 and CD86, the costimulatory molecules necessary for T cell activation. The flow cytometry results and statistical analyses are shown in Figure 2C. SZU251 and SZU251 + MUC1 increased the levels of CD40, CD80 and CD86, while SZU251 + MUC1 exhibited an even better stimulatory capacity than SZU251. However, significant differences between SZU251 and SZU251 + MUC1 could be observed for CD40 and CD86 but not CD80, which may be due to the different sensitivity of the costimulatory molecules. Moreover, SZU251 + MUC1 promoted the expression of the costimulatory molecules basically in a dose-dependent manner, similar to the results of cytokine levels (Figure S4).

### 2.3. SZU251 + MUC1 + Al Inhibited 4T1 Mouse Breast Tumor Growth in the Prophylactic Schedule

**Figure 3:**
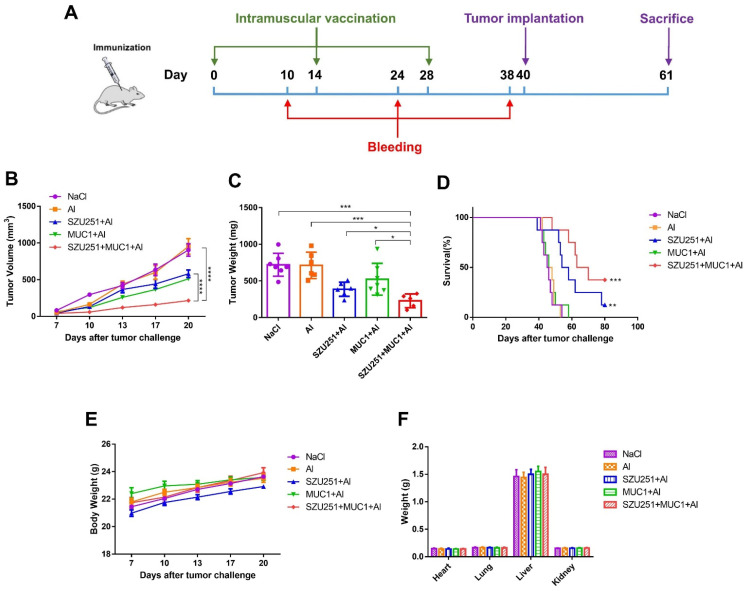
SZU251 + MUC1 + Al inhibited 4T1 mouse breast tumor growth after three immunizations in the prophylactic schedule. (**A**) Schematic diagram illustrating the prophylactic vaccination. (**B**) Tumor growth curves were measured twice a week until day 21 after tumor implantation. (**C**) Tumor weights were determined at the time of sacrifice. (**D**) Survival curves of the tumor-bearing mice. (**E**) Body weights were measured twice a week until day 21 after tumor implantation. (**F**) Weights of the major organs (hearts, lungs, livers and kidneys) were determined at the time of sacrifice. Data are presented as the mean ± SE; *n* = 5–8 mice/group. * *p* < 0.05, ** *p* < 0.01, *** *p* < 0.001, **** p < 0.0001.

The antitumor effects of the vaccine candidates were further examined in a mouse breast cancer model with the prophylactic schedule. In brief, each mouse was intramuscularly administered with vaccines every two weeks for a total of three times and then subcutaneously challenged with 4T1 cells. Twenty-one days after the tumor inoculation, the mice were sacrificed and the tumors were collected (Figure 3A). The tumor volumes, tumor weights and representative images of the tumors are illustrated in Figure 3B,C and Figure S5. All of the vaccine candidates displayed antitumor effects compared to the saline control (NaCl), except the aluminum adjuvant alone (Al). Notably, the tumor volumes and tumor weights when treated with SZU251 + MUC1 + Al were much lower than when treated with SZU251 + Al or MUC1 + Al. The results also showed that SZU251 + MUC1 + Al markedly improved the long-term survival rate compared to the other groups during a 70-day observation period (Figure 3D).

During the experiment, all of the mice appeared healthy with no visible signs of pain, distress or discomfort. No significant differences in the body weights or the weights of the major organs could be detected between the groups (Figure 3E,F), indicating that the vaccines were well tolerated at these doses. HE staining of the major organs was carried out, and no serious structural and pathological changes were observed in all of the groups (Figure S6). Blood tests were performed, and the results are shown in Figure S7 and Table 1. There were some individual slight decreases in the WBC and PLT counts in the vaccine groups that were still considered within the normal range, while other parameters (RBC, HGB, HCT and MCV) displayed no significant abnormality.

### 2.4. SZU251 + MUC1 + Al Induced Tumor-Specific Immune Responses in the Prophylactic Schedule

**Figure 4:**
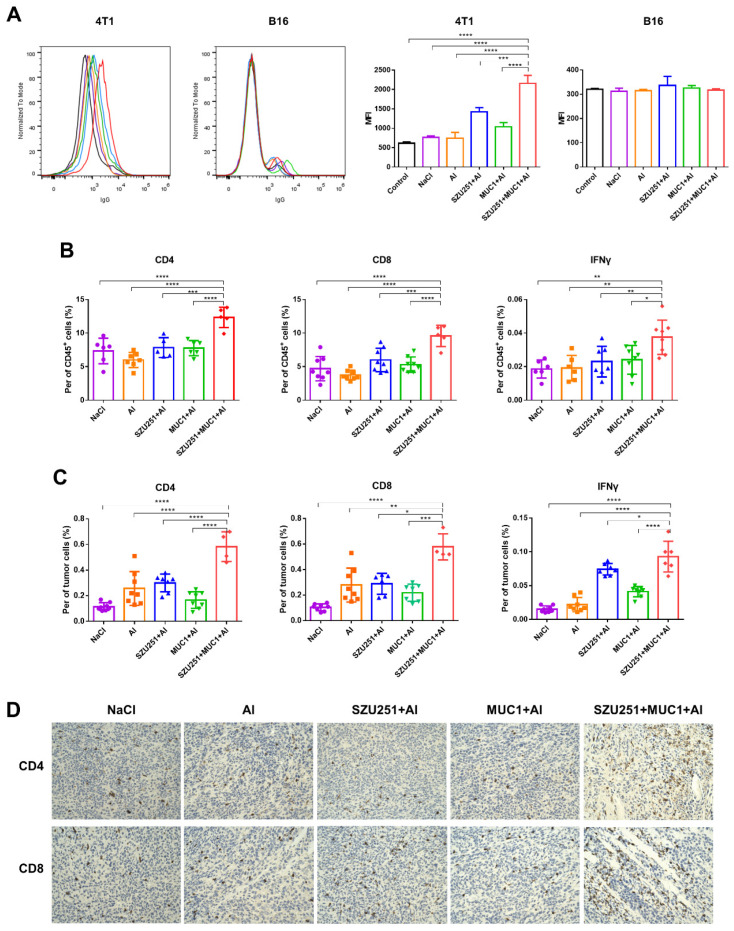
SZU251 + MUC1 + Al induced tumor-specific immune responses in the prophylactic schedule. (**A**) Specific anti-MUC1 antibody of the serum samples was tested on 4T1 and B16 cells using flow cytometry. NaCl (purple), Al (orange), SZU251 + Al (blue), MUC1 + Al (green) and SZU251 + MUC1 + Al (red). (**B**) Percentages of CD3^+^ CD4^+^ T cells, CD3^+^ CD8^+^ T cells and CD3^+^ CD8^+^ IFNγ^+^ T cells in splenocytes were measured using flow cytometry. (**C**) Percentages of CD3^+^ CD4^+^ T cells, CD3^+^ CD8^+^ T cells and CD3^+^ CD8^+^ IFNγ^+^ T cells in TILs were measured using flow cytometry. (**D**) Representative IHC staining for CD4 and CD8 of the tumor tissues in the prophylactic schedule (200× magnification). Data are presented as the mean ± SE; *n* = 5–8 mice/group. * *p* < 0.05, ** *p* < 0.01, *** *p* < 0.001, **** p < 0.0001.

Serum antibody titers against MUC1 were judged by the ELISA method to prove the impacts of the vaccinations on humoral immunity (Table 2). As expected, no antibody could be found in the vaccine groups without the MUC1 component (NaCl, Al and SZU251 + Al), while MUC1 + Al elicited total IgG, IgG1, IgG2a and IgM responses. SZU251 + MUC1 + Al prominently elevated the antibody titers to much higher levels than MUC1 + Al did. Meanwhile, the more times vaccinations were administered, the more potent antibody responses we could detect. In addition, no significant changes of IgG2a and IgM could be found between day 24 and day 38 in the SZU251 + MUC1 + Al group. It is supposed that these types of antibodies reached a plateau after two vaccinations. To validate whether the serum samples could recognize the native MUC1 antigen on cancer cells, binding of the serum samples to MUC1-expressing 4T1 cells was examined by flow cytometry (Figure 4A). The serum samples from SZU251 + MUC1 + Al reacted much more strongly with 4T1 cells than the other vaccine candidates did. B16 cells, in which the expression of MUC1 antigen is relatively low, were used as a negative control, and no obvious binding was observed.

Cellular immune responses were determined by analyzing CD4^+^ and CD8^+^ T cells in the spleens, while activated CD8^+^ T cells were further identified by intracellular IFNγ staining. As shown in Figure 4B, SZU251 + MUC1 + Al boosted the percentages of total CD4^+^, total CD8^+^ and CD8^+^ IFNγ^+^ cells more effectively than any other vaccines did. T cells were also evaluated in the tumors to study whether tumor-specific T cells were recruited to the tumor sites. SZU251 + MUC1 + Al increased the percentages of total CD4^+^, total CD8^+^ and CD8^+^ IFNγ^+^ cells to the highest levels, which is consistent with the results in the spleens (Figure 4C). IHC staining was performed in the tumor tissues, and the results are shown in Figure 4D. The differences in CD4^+^ and CD8^+^ T cells were negligible in the NaCl, Al, SZU251 + Al and MUC1 + Al groups, while CD4^+^ and CD8^+^ T cells infiltration was significantly promoted by SZU251 + MUC1 + Al.

### 2.5. SZU251 + MUC1 + Al Inhibited 4T1 Mouse Breast Tumor Growth in the Therapeutic Schedule

**Figure 5:**
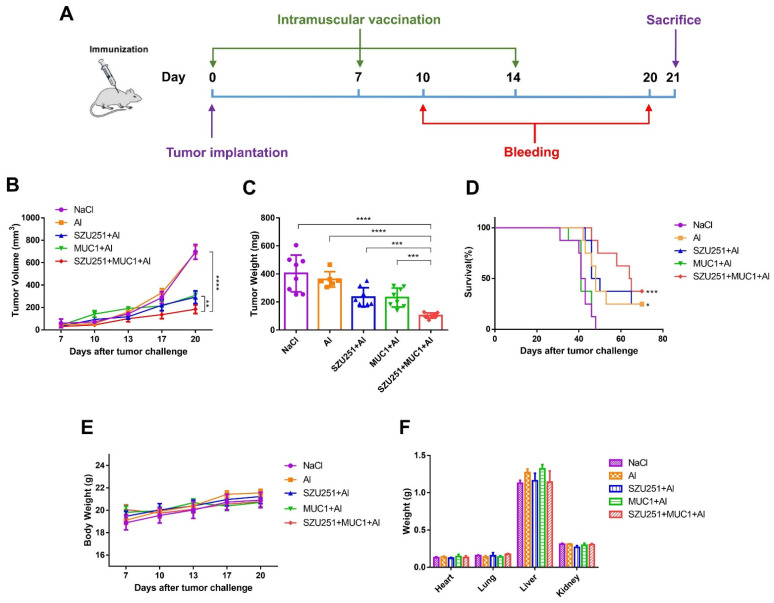
SZU251 + MUC1 + Al inhibited 4T1 mouse breast tumor growth after three immunizations in the therapeutic schedule. (**A**) Schematic diagram illustrating the therapeutic vaccination. (**B**) Tumor growth curves were measured twice a week until day 21 after tumor implantation. (**C**) Tumor weights were determined at the time of sacrifice. (**D**) Survival curves of the tumor-bearing mice. (**E**) Body weights were measured twice a week until day 21 after tumor implantation. (**F**) Weights of the major organs (hearts, lungs, livers and kidneys) were determined at the time of sacrifice. Data are presented as the mean ± SE; *n* = 5–8 mice/group. * *p* < 0.05, ** *p* < 0.01, *** *p* < 0.001, **** p < 0.0001.

The antitumor effects of the vaccine candidates were also examined in a mouse breast cancer model with a therapeutic schedule. In brief, each mouse was first subcutaneously challenged with 4T1 cells and then intramuscularly administered with vaccines once a week for a total of three times. Twenty-one days after the tumor inoculation, the mice were sacrificed and the tumors were collected (Figure 5A). The tumor volumes, tumor weights and representative images of the tumors are illustrated in Figure 5B,C and Figure S8. Long-term survival curves were recorded as well, as shown in Figure 5D. SZU251 + MUC1 + Al evidently suppressed tumor growth and promoted the survival rate compared to the other vaccine groups. In addition, as shown in Figure 5B,D, MUC1 + Al was effective against the primary tumor growth but not the death of the mice, which might be due to the lung metastasis in the 4T1 model. Hence, SZU251 probably enhanced the antitumor effects of MUC1 + Al.

Toxicity was thoroughly determined, and the vaccines were well tolerated at these doses in the therapeutic schedule. There were no significant differences of the body weights or the weights of the major organs, and no structural or pathological changes of the major organs in all of the vaccine groups (Figure 5E,F and Figure S9). No significant abnormality could be detected in all of the parameters (WBC, RBC, HGB, HCT, MCV and PLT) in blood tests (Figure S10 and Table 3).

### 2.6. SZU251 + MUC1 + Al Induced Tumor-Specific Immune Responses in the Therapeutic Schedule

**Figure 6:**
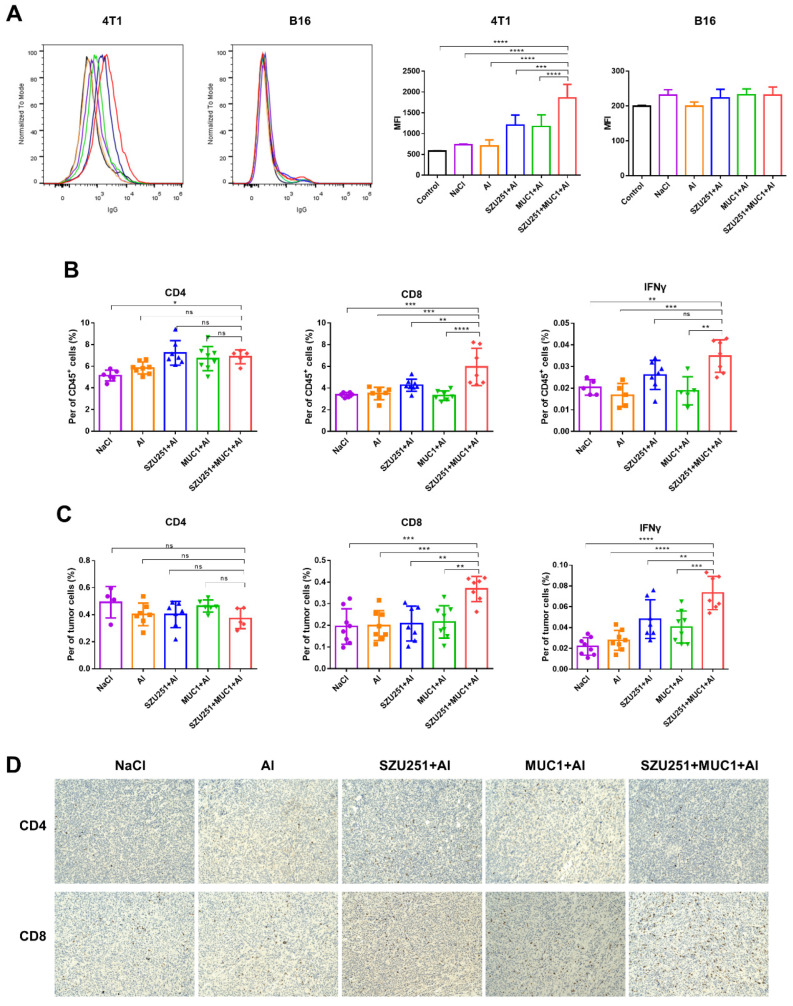
SZU251 + MUC1 + Al induced tumor-specific immune responses in the therapeutic schedule. (**A**) Specific anti-MUC1 antibody of the serum samples was tested on 4T1 and B16 cells using flow cytometry. NaCl (purple), Al (orange), SZU251 + Al (blue), MUC1 + Al (green) and SZU251 + MUC1 + Al (red). (**B**) Percentages of CD3^+^ CD4^+^ T cells, CD3^+^ CD8^+^ T cells and CD3^+^ CD8^+^ IFNγ^+^ T cells in splenocytes were measured using flow cytometry. (**C**) Percentages of CD3^+^ CD4^+^ T cells, CD3^+^ CD8^+^ T cells and CD3^+^ CD8^+^ IFNγ^+^ T cells in TILs were measured using flow cytometry. (**D**) Representative IHC staining for CD4 and CD8 of the tumor tissues in the therapeutic schedule (200× magnification). Data are presented as the mean ± SE; *n* = 5–8 mice/group. * *p* < 0.05, ** *p* < 0.01, *** *p* < 0.001, **** p < 0.0001.

Humoral immune responses of the vaccinations are displayed as serum antibody titers against MUC1 judged by the ELISA method (Table 4). MUC1 + Al and SZU251 + MUC1 + Al remarkably elicited total IgG, IgG1, IgG2a and IgM responses, while SZU251 + MUC1 + Al was much more potent than MUC1 + Al. The serum antibody titers from 20 days after the first immunization were higher than those from 10 days after the first immunization. Binding of the serum samples to the tumor cells was examined by flow cytometry (Figure 6A). The serum samples from SZU251 + MUC1 + Al reacted strongly to 4T1 cells with high MUC1 expression but not B16 cells with low MUC1 expression.

Cellular immune responses to the vaccinations were determined by analyzing CD4^+^ and CD8^+^ T cells in the spleens and the tumors, while activated CD8^+^ T cells were further identified by intracellular IFNγ staining (Figure 6B,C). SZU251 + MUC1 + Al boosted the percentages of total CD8^+^ and CD8^+^ IFNγ^+^ cells both in the spleens and the tumors more effectively than any other vaccines. However, CD4^+^ T cells were not impacted as much as CD8^+^ T cells, where SZU251 + MUC1 + Al exhibited higher percentages of total CD4^+^ T cells only in the spleens compared to the saline control (NaCl). IHC staining of the tumor tissues proved that the infiltration of CD8^+^ T cells, but not CD4^+^ T cells, was significantly promoted by SZU251 + MUC1 + Al (Figure 6D).

## 3. Discussion

Breast cancer, especially triple-negative breast cancer (TNBC), is a biologically and clinically heterogeneous disease which has been considered a major cause of morbidity and mortality due to its aggressive behavior and poor prognosis [15]. Traditional treatments, including surgery and chemotherapy, are not satisfactory enough for all of the patients. Decades of investigations on breast cancer have led to the development of new therapeutic options in recent years, such as immune checkpoint inhibitors, antibody–drug conjugates, tumor vaccines and other promising drug combinations.

Tumor vaccines come into effect by training the immune system to attack the cells that contain the tumor-associated antigens (TAAs) in the vaccines. A protein with an abnormal structure produced by tumor cells due to mutation can be an effective TAA, and abnormal glycosylation is an example of the mutation. MUC1 is a typical glycosylated tumor antigen expressed on the surface of cancer cells. Compared to normal tissues, the expression levels and glycosylation patterns of MUC1 are highly dissimilar in tumor tissues [16]. In a phase 2b/3 trial for advanced non-small-cell lung cancer, TG4010 (a modified vaccinia Ankara expressing MUC1) plus chemotherapy showed a significant improvement in progression-free survival compared to a placebo plus chemotherapy [17]. MUC1-specific T cells and antibodies are also identified in breast cancer patients, and MUC1-specific immunity is beneficial in the treatment of breast cancer [18,19]. It was reported that an MUC1 mRNA nano-vaccine induced a strong, antigen-specific, in vivo cytotoxic T lymphocyte response against TNBC cells [20]. However, the MUC1 antigen itself does not have enough immunogenicity to arouse efficient immune responses. Therefore, we introduced a small-molecule TLR7 agonist as a promising adjuvant for the activation of a broad spectrum of APCs, including different types of DCs [21]. The novel TLR7 agonist, SZU251, was synthesized by our group for the first time and exerted stronger effects on TLR7 activation than imiquimod (Figure 1B and Figure S3). Moreover, after mixing with MUC1, SZU251 + MUC1 maintained similar immunostimulatory effects as SZU251. In agreement with the previous studies, SZU251 + MUC1 caused a rapid induction of common cytokines such as IL6, TNFα and IFNγ in mouse BMDCs and spleen lymphocytes in vitro (Figure 2A,B), favoring Th1-mediated immune responses and cytotoxic T cells acting on cancer cells [22]. SZU251 + MUC1 also promoted the expression of costimulatory molecules (CD40, CD80 and CD86) in mouse BMDCs in a concentration-dependent manner (Figure 2C and Figure S4), inferring the maturation of BMDCs and the activation of T cells [23]. Additionally, SZU251 is an isothiocyanate (-N=C=S) derivative of our previous TLR7 agonist, SZU101, which could be easily coupled to the amino groups of the proteins. In future studies, we will conjugate the TLR7 agonists to other tumor-associated antigens, especially macromolecular protein antigens, where SZU251 could be more convenient for conjugation than SZU101.

An outstanding tumor vaccine should trigger cellular and humoral immunity simultaneously to elicit robust and long-lasting immune responses [24]. We have constructed several tumor vaccines by conjugating TLR7 agonists and TAAs and have proven the antitumor effects of the vaccines in previous studies. Nevertheless, the humoral responses of the vaccines are still not satisfactory, even with the introduction of our TLR7 agonists [8,14]. The aluminum adjuvant is well known for the promotion of humoral immune responses by adsorbing antigens and altering their immunological properties [25]. Therefore, we constructed a novel vaccine (SZU251 + MUC1 + Al) with three components in this study. SZU251 and MUC1 were first mixed at a molar ratio of 3:1 to form SZU251 + MUC1, and then, SZU251 + MUC1 was absorbed on the aluminum adjuvant to form SZU251 + MUC1 + Al (Figure 1C). Almost all of the experiments in this study displayed that the aluminum adjuvant (Al) alone exerted no effects on tumor inhibition or immune stimulation compared to the control. Therefore, we only included the SZU251 + MUC1 + Al group and not the SZU251 + MUC1 group, and the efficacy of SZU251 + MUC1 + Al could be determined compared to either the control or Al alone. After three immunizations, a tumor challenge model was generated in BALB/c mice via the implantation of mouse 4T1 TNBC cells (Figure 3A). SZU251 + MUC1 + Al showed the best prophylactic effects with suppression of the tumor growth and extension of the survival time of the mice (Figure 3B,D and Figure S5). It is reported that functional activation of TLR7 might lead to the loss of body weight and a decrease in platelet counts, and chronic TLR7 signaling drives anemia through the differentiation of specialized hemophagocytes [26]. In our toxicity studies, except for a slight decrease in the WBC count, no significant differences could be detected between the groups in the body weights of the mice, the weights and HE staining of the major organs and the results of the blood tests (Figure 3E,F, Figures S6 and S7). Thus, the safety of SZU251 + MUC1 + Al was displayed in the prophylactic schedule.

As for the humoral responses, SZU251 + MUC1 + Al induced a substantial increase in antibody titers for MUC1-specific IgG, IgG1, IgG2a and IgM in a time-dependent manner (Table 2). IgG2a and IgG1 represent immune Th1 and Th2 tendencies, respectively. Th1 is the most important helper cell type in cancer immunity, involved in tumor cell killing by secreting cytokines that activate tumor cell surface death receptors and induce epitope spreading [27,28]. Our results demonstrated that after three immunizations, the ratio of IgG2a to IgG1 of SZU251 + MUC1 + Al was the highest compared to the other groups, indicating the tendency of Th1. IgM is also part of the first line of immunological defense. Natural IgM is associated with the recognition and removal of cancerous cells, and some IgM antibodies are used in the diagnosis and treatment of breast cancer [29]. Furthermore, the recognition of native MUC1 antigen on 4T1 cells by the antibodies was verified by flow cytometry (Figure 4A).

As for the cellular responses, lymphocytes in the spleens and the tumors were analyzed by flow cytometry. The main types of lymphocytes in cell-mediated immunity are CD4^+^ and CD8^+^ T cells, which play a critical role in the induction of an effective immune response against tumors [30]. As shown in Figure 4B, significant increases in CD4^+^, CD8^+^ and activated CD8^+^ T cells were detected in the spleens treated with SZU251 + MUC1 + Al. CD4^+^, CD8^+^ and activated CD8^+^ T cell infiltration to the tumor microenvironment was also demonstrated by flow cytometry and IHC staining (Figure 4C,D).

In addition to the above results of the vaccines in the prophylactic schedule, we explored their benefits in the therapeutic schedule. BALB/c mice were first implanted with mouse 4T1 TNBC cells and then treated with the vaccines three times. As shown in Figure 5 and Figures S8–S10, SZU251 + MUC1 + Al displayed high efficacy and low toxicity in the treatment of breast cancer. Humoral and cellular immune responses were determined in the therapeutic schedule, with similar effects as the prophylactic schedule (Figure 6 and Table 4). Only the promotion of CD4^+^ T cells by SZU251 + MUC1 + Al was weak, which may be due to the limited time of drug administration with low recognition of epitopes [31].

In conclusion, a novel vaccine for breast cancer was developed with three components: a small-molecule TLR7 agonist (SZU251), an MUC1-related peptide (MUC1) and an aluminum adjuvant (Al). Our results demonstrated that SZU251 + MUC1 + Al was effective and safe against a tumor expressing the MUC1 antigen in both prophylactic and therapeutic schedules. Incorporation of the TLR7 agonist and the aluminum adjuvant induced non-specific immune responses and substantially enhanced the specific humoral and cellular immune responses to the MUC1 antigen in the mice. Therefore, our vaccine candidate may have beneficial effects in the prevention and treatment of breast cancer patients.

## 4. Materials and Methods

### 4.1. Compounds

The MUC1 peptide used in the present study was obtained by the solid phase method using an Fmoc strategy (ChinaPeptides Co., Ltd., Shanghai, China).

SZU251 was synthesized by our laboratory with the following three steps. First, 1 g SZU101, 540 mg 2-(2-(2-(2-azidoethoxy)ethoxy)ethoxy)ethan-1-amine, 460 mg HOBT, 660 mg EDC and 1200 μL DIPEA were dissolved in 3 mL DMF, and the reaction was monitored by LC-MS at room temperature overnight. The reaction was purified by HPLC to yield 700 mg A1 as a white solid with 48.3% yield. Second, 500 mg A1 and 50 mg Pd/C (10%) were dissolved in 5 mL of MeOH, evacuated and passed through H_2_, and the reaction was monitored by LC-MS for 2 h at room temperature. After the reaction, Pd/C was removed by filtration, and the product A2 was obtained by spin-drying with MeOH. Third, 500 mg A2, 130 mg CS_2_ and 230 μL TEA were dissolved in 2 mL DMF and reacted overnight at room temperature; then, 310 mg TsCl was added, and the reaction was monitored by LC-MS for 5 h at room temperature. After the reaction, it was purified by HPLC, and 320 mg of white solid (SZU251) was obtained at 60% yield.

SZU251: ^1^H NMR (500 MHz, DMSO-d6) δ 10.08 (s, 1H), 8.28 (t, J = 5.8 Hz, 1H), 7.86 (t, J = 5.5 Hz, 1H), 7.23 (d, J = 8.1 Hz, 2H), 7.18 (d, J = 8.1 Hz, 2H), 6.50 (s, 2H), 4.82 (s, 2H), 4.29–4.23 (d, J = 5.0 Hz, 2H), 4.20 (d, J = 5.8 Hz, 2H), 3.80 (t, J = 5.1 Hz, 2H), 3.61 (t, J = 5.1 Hz, 2H), 3.60–3.55 (m, 4H), 3.55–3.46 (m, 7H), 3.38 (t, J = 5.9 Hz, 2H), 3.27 (s, 3H), 3.17 (q, J = 5.8 Hz, 2H), 2.33 (s, 4H). ^13^C NMR (125 MHz, DMSO-d6) δ 171.36, 171.28, 159.81, 152.22, 149.13, 147.72, 138.78, 135.60, 127.48 (2C), 127.34 (2C), 98.33, 70.23, 69.77, 69.75, 69.63, 69.57, 69.10, 68.42, 65.26, 58.07, 45.04, 42.13, 41.75, 40.11, 38.54, 30.73, 30.71.

An aluminum adjuvant (Alhydrogel^®^ adjuvant 2%, InvivoGen, San Diego, CA, USA) containing 8% of the total volume was added to the antigen solution (SZU251, MUC1 or SZU251 + MUC1) and mixed well at 1000 rpm at room temperature for 2 h. Here, for SZU251 + MUC1 + Al, SZU251 and MUC1 were first mixed at a molar ratio of 3:1 and then mixed thoroughly with the aluminum adjuvant.

### 4.2. Mice and Cell Lines

For the experiments, 4T1 mouse breast cancer cells, mouse BMDCs and mouse spleen lymphocytes were cultured in RPMI-1640 medium supplemented with 10% FBS, 100 µg/mL penicillin and 100 µg/mL streptomycin (Gibco, Waltham, MA, USA) at 37 °C in a humidified atmosphere with 5% CO_2_. All experiments were performed using mycoplasma-free cells.

Female 4-week-old BALB/c mice (weight 15–20 g) were purchased from the Guangdong Medical Laboratory Animal Center, China. All mice were housed under constant specific pathogen-free laboratory conditions at 18–22 °C, 50–60% humidity, 12 h of light/dark cycling and with ad libitum access to water and food. The protocol for animal experiments was approved by the Ethics Committee of Experimental Animals of Shenzhen University, Shenzhen, China.

### 4.3. HEK-Blue Assay

HEK-Blue hTLR7 and hTLR8 cells (InvivoGen) were cultured in DMEM supplemented with 10% FBS, blasticidin, zeocin and normocin (InvivoGen). The cells (2.5 × 10^4^/well) were seeded into 96-well plates and then treated with the compounds in HEK-Blue Detection medium (InvivoGen) for 14 h. The activation of TLR7 and TLR8 was assessed by a microplate reader (BioTek, Winooski, VT, USA).

### 4.4. BMDCs Preparation

All femurs and tibias of the mice were surgically removed. The ends of the bones were cut off with scissors, and the bones were repeatedly rinsed out into the culture dish. The bone marrow suspension was collected, filtered with a 200-micron nylon mesh and centrifuged at 1200 rpm for 5 min. Then, the obtained mouse bone marrow cells were adjusted to 2 × 10^5^ cells/mL and seeded into 100 mm bacterial culture dishes (Petri dish), and recombinant mouse GM-CSF (20 ng/mL) was added. On day 3, 10 mL of complete culture medium containing 20 ng/mL GM-CSF was added to the culture dish. On day 6 and day 8, the old culture medium was collected in half volume, centrifuged and resuspended with complete culture medium containing 20 ng/mL GM-CSF; then, the cell suspension was put back into the original dish. On day 10, the cells could be collected as BMDCs.

### 4.5. BMDCs Maturation

The prepared BMDCs (1 × 10^6^ cells/mL/well) were cultured with the compounds for 24 h at 37 °C and stained with anti-mouse CD11c-PerCP/Cy5.5, CD40-APC, CD80-FITC and CD86-BV650 antibodies (BioLegend, San Diego, CA, USA) at room temperature for 30 min in the dark. The cells were analyzed using an Attune NxT Flow Cytometer (Invitrogen, Waltham, MA, USA).

### 4.6. Spleen Lymphocytes Preparation

The spleens of the mice were surgically removed and ground with 4 mL of mouse lymphocyte separation solution. After centrifugation, the middle layer of the liquid was harvested and washed once by centrifugation. Then, 1 mL of lysis solution was used to lyse the erythrocytes for 1 min, and the remaining cells were collected as mouse spleen lymphocytes after washing with PBS.

### 4.7. Analysis of Cytokine Levels by ELISA

The prepared spleen lymphocytes and BMDCs (1 × 10^6^ cells/mL/well) were cultured with the compounds for 24 h. The total cytokine levels generated by the vaccine candidates were evaluated using ELISA kits (IFNγ, TNFα, IL6 and IL12) (Invitrogen) as per the manufacturer’s instructions. Briefly, high-binding 96-well plates were coated with capture antibodies at 4 °C overnight. Then, the coated plates were blocked by diluent buffer, added with the samples and incubated for 2 h at room temperature. Following incubation with the detection antibodies and Avidin-HRP for 1 h at room temperature, the plates were incubated with 3,3′,5,5′-tetramethylbenzidine (TMB) substrate solution for 15–20 min and then sulfuric acid. Absorbance was measured at 450 nm with a microplate reader (BioTek, Winooski, VT, USA).

### 4.8. Immunization of Mice

Five groups of female BALB/c mice were examined with different vaccine candidates: (i) normal saline (NaCl); (ii) Alhydrogel adjuvant 2% (Al); (iii) SZU251 (57 μg) with Alhydrogel adjuvant (SZU251 + Al); (iv) MUC1 (100 μg) with Alhydrogel adjuvant (MUC1 + Al); (v) SZU251 + MUC1 (157 μg) with Alhydrogel adjuvant (SZU251 + MUC1 + Al). In the prophylactic schedule, mice were immunized by intramuscular injection into the right hind leg on day 0, day 14 and day 28. The mice were bled on day 10, day 24 and day 38 after boost immunizations. In the therapeutic schedule, mice were immunized by intramuscular injection into the right hind leg on day 0, day 7 and day 14. The mice were bled on day 10 and day 20 after boost immunizations. Mouse blood samples were placed at 4 °C overnight and centrifuged at 3000 rpm for 10 min. The supernatant was collected as serum and stored at −80 °C before use.

### 4.9. Analysis of Antibody Titers and Subtypes by ELISA

The antibody titers and antibody isotypes generated by the vaccine candidates were measured by ELISA. The MUC1 peptide as an antigen was dissolved in a NaHCO_3_/Na_2_CO_3_ buffer with a final concentration of 1 μg/mL. Next, 96-well plates were coated with MUC1 and incubated at 4 °C overnight. Then, the coated plates were blocked with dilution buffer and incubated with the serially diluted serum samples at 37 °C for 2 h. Following incubation with either HRP-linked goat anti-mouse antibody IgG, IgM, IgG1 or IgG2a (Immunoway, Plano, TX, USA) (1:5000 dilution) at 37 °C for 1 h, the plates were incubated with TMB substrate solution for 15 min and then sulfuric acid. Absorbance was recorded at 450 nm with a microplate reader (BioTek). Antibody titer was defined as the maximum serum dilution when the absorption ratio of the experimental group to the negative control group was ≥2.0.

### 4.10. Analysis of Antigen Recognition Ability

Serum samples collected after immunizations were diluted 10-fold and incubated with a 4T1 or B16 single cell suspension on ice for 1 h in the dark. Next, the cells were washed and incubated with goat anti-mouse IgG γ-chain-specific antibody conjugated to fluorescein isothiocyanate (IgG-FITC; Biolegend) on ice for 1 h in the dark. Cells were analyzed using an Attune NxT Flow Cytometer (Invitrogen) and FlowJo X 10.0.7 R2 software (BD Biosciences, Franklin Lakes, NJ, USA).

### 4.11. Evaluation of Antitumor Effects in Vaccinated Mice

For the prophylactic schedule, on day 40, mice were subcutaneously implanted with 4T1 cell suspension (3 × 10^5^ cells). Tumor size was measured twice a week, and tumor volume was calculated according to the following equation: Volume = L × W^2^/2, where L (length) and W (width) are the long and short axes of the tumor, respectively. On day 61, the mice were sacrificed with carbon dioxide inhalation to reduce animal pain, and tumors and spleens were surgically dissected, weighed and photographed. For the therapeutic schedule, mice were implanted with 4T1 cell suspension on day 0, and tumors and spleens were removed on day 21. In both sets of experiments, an additional 40 mice were used to assess long-term survival until their natural death or until the tumor size reached 20 mm in diameter.

### 4.12. Detection of T Lymphocytes

Spleens and tumors were harvested at the time of sacrifice. Spleens were ground and red blood cells were lysed. Tumors were ground and digested with collagenase A and DNase I at 37 °C for 40 min. Single cell suspensions of splenocytes and tumor cells (2 × 10^6^ cells/spleen, 5 × 10^6^ cells/tumor) were stained with anti-mouse CD45-BV605, CD3-FITC, CD4-PE and CD8-PerCP/Cy5.5 antibody (cell surface antibody) (BioLegend), protecting from light at 4 °C for 30 min. The cells were fixed and permeabilized for intracellular staining (Fixation/Permeabilization Solution kit, eBioscience, San Diego, CA, USA) and stained with anti-mouse IFNγ-BV421 antibody (intracellular antibody) (BioLegend) at room temperature for 30 min in the dark. Subsequently, cells were analyzed using an Attune NxT Flow Cytometer (Invitrogen) and FlowJo v10 software.

### 4.13. Immunohistochemistry (IHC) Staining

Tumor tissues were fixed in 4% paraformaldehyde, embedded in paraffin and transversely cut. Sections were stained with anti-mouse CD4 and CD8 primary antibody (Abcam, Cambridge, UK) at 4 °C overnight and HRP-conjugated secondary antibody (Abcam) at room temperature for 1 h. Additionally, 3′-3-diaminobenzidine (DAB) substrate was used for color development. Cell nuclei were further stained with hematoxylin. The sections were dehydrated, sealed and photographed with an EVOS fluorescence microscope (Invitrogen) at 200× magnification.

### 4.14. Toxicity Studies

After tumor implantation, the body weights of the mice were measured twice a week. At the time of sacrifice, blood samples and major organs (hearts, livers, lungs and kidneys) were collected. The weights of the major organs were measured, and pathological changes of the major organs were observed by HE staining. Routine blood examination was performed using a blood test instrument (Mindray, Shenzhen, China), where the changes in white blood cell (WBC) count, red blood cell (RBC) count, hemoglobin (HGB), hematocrit (HCT), mean corpuscular volume (MCV) and platelet (PLT) were recorded.

### 4.15. Statistical Analysis

Statistical analysis was performed using the GraphPad Prism5 software. A two-tailed *t*-test and one-way ANOVA test were performed for comparisons between two and more groups, respectively. Statistical analysis of the data from the tumor growth curves was performed using a two-way ANOVA test. Data are presented as the mean ± SE. Differences were considered statistically significant when *p* < 0.05.

## Figures and Tables

**Table 1:** Blood routine examination of the mice in the prophylactic schedule.

Group	NaCl	Al	SZU251 + Al	MUC1 + Al	SZU251 + MUC1 + Al
WBC (10^9^/L)	30.05 ± 4.96	28.70 ± 9.49	27.68 ± 7.59	18.63 ± 5.74	13.85 ± 3.24
RBC (10^12^/L)	11.17 ± 0.56	11.64 ± 0.95	11.74 ± 0.69	11.07 ± 0.71	11.40 ± 0.49
HGB (g/L)	175.20 ± 7.73	186.50 ± 9.33	181.25 ± 5.56	171.20 ± 10.69	169.00 ± 8.69
HCT (%)	55.40 ± 2.42	57.45 ± 4.62	55.24 ± 3.23	55.36 ± 3.23	54.12 ± 2.91
MCV (fL)	49.66 ± 0.47	49.40 ± 0.86	47.11 ± 0.98	50.08 ± 1.00	47.52 ± 1.08
PLT (10^9^/L)	1022.67 ± 168.50	1045.50 ± 258.30	957.50 ± 208.63	795.50 ± 138.85	809.00 ± 43.70

Abbreviations: WBC, white blood cell; RBC, red blood cell; HGB, hemoglobin; HCT, hematocrit; MCV, mean corpuscular volume; PLT, platelet.

**Table 2:** Anti-MUC1 antibody titers determined by ELISA after three immunizations with vaccine candidates in the prophylactic schedule.

Antibody		Titer				
		NaCl	Al	SZU251 + Al	MUC1 + Al	SZU251 + MUC1 + Al
IgG	Day 10	0	0	0	1000	16,000
	Day 24	0	0	0	32,000	1,024,000
	Day 38	0	0	0	256,000	2,048,000
IgG1	Day 10	0	0	0	0	0
	Day 24	0	0	0	8000	64,000
	Day 38	0	0	0	8000	128,000
IgG2a	Day 10	0	0	0	0	32,000
	Day 24	0	0	0	16,000	2,048,000
	Day 38	0	0	0	16,000	2,048,000
IgM	Day 10	0	0	0	0	16,000
	Day 24	0	0	0	4000	128,000
	Day 38	0	0	0	16,000	128,000

Antibody titer was defined as the maximum serum dilution when the absorption ratio of the experimental group to the negative control group was ≥2.0.

**Table 3:** Blood routine examination of the mice in the therapeutic schedule.

Group	NaCl	Al	SZU251 + Al	MUC1 + Al	SZU251 + MUC1 + Al
WBC (10^9^/L)	13.33 ± 0.78	9.68 ± 5.12	15.48 ± 5.31	12.60 ± 2.52	15.30 ± 3.48
RBC (10^12^/L)	9.85 ± 0.40	10.00 ± 0.94	9.57 ± 0.33	8.70 ± 0.46	9.34 ± 0.81
HGB (g/L)	144.80 ± 4.15	142.00 ± 7.00	144.40 ± 7.54	134.00 ± 5.29	140.20 ± 9.40
HCT (%)	44.83 ± 1.35	45.93 ± 3.95	43.86 ± 1.95	40.56 ± 1.70	43.34 ± 3.67
MCV (fL)	46.92 ± 0.47	46.03 ± 0.76	45.77 ± 0.21	46.98 ± 0.89	45.87 ± 0.35
PLT (10^9^/L)	739.40 ± 128.80	633.80 ± 288.80	1056.00 ± 214.99	933.40 ± 188.03	956.75 ± 231.80

Abbreviations: WBC, white blood cell; RBC, red blood cell; HGB, hemoglobin; HCT, hematocrit; MCV, mean corpuscular volume; PLT, platelet.

**Table 4:** Anti-MUC1 antibody titers determined by ELISA after three immunizations with vaccine candidates in the therapeutic schedule.

Antibody		Titer				
		NaCl	Al	SZU251 + Al	MUC1 + Al	SZU251 + MUC1 + Al
IgG	Day 10	0	0	0	32,000	64,000
	Day 20	0	0	0	128,000	1,024,000
IgG1	Day 10	0	0	0	0	0
	Day 20	0	0	0	32,000	32,000
IgG2a	Day 10	0	0	0	32,000	64,000
	Day 20	0	0	0	32,000	1,024,000
IgM	Day 10	0	0	0	8000	128,000
	Day 20	0	0	0	32,000	256,000

Antibody titer was defined as the maximum serum dilution when the absorption ratio of the experimental group to the negative control group was ≥2.0.

## Data Availability

The data needed to evaluate the conclusions are present in the paper and/or the Supplementary Materials.

## Supplementary Materials

The following supporting information can be downloaded at: https://www.mdpi.com/article/10.3390/ijms232315130/s1.

## References

[1] Mokhtari Y., Pourbagheri-Sigaroodi A., Zafari P., Bagheri N., Ghaffari S.H., Bashash D. (2021). Toll-like receptors (TLRs): An old family of immune receptors with a new face in cancer pathogenesis. J. Cell. Mol. Med..

[2] Petes C., Odoardi N., Gee K. (2017). The Toll for Trafficking: Toll-Like Receptor 7 Delivery to the Endosome. Front. Immunol..

[3] Ding R., Jiao A., Zhang B. (2022). Targeting toll-like receptors on T cells as a therapeutic strategy against tumors. Int. Immunopharmacol..

[4] Wan D., Que H., Chen L., Lan T., Hong W., He C., Yang J., Wei Y., Wei X. (2021). Lymph-Node-Targeted Cholesterolized TLR7 Agonist Liposomes Provoke a Safe and Durable Antitumor Response. Nano Lett..

[5] Zhang H., Tang W.L., Kheirolomoom A., Fite B.Z., Wu B., Lau K., Baikoghli M., Raie M.N., Tumbale S.K., Foiret J. (2021). Development of thermosensitive resiquimod-loaded liposomes for enhanced cancer immunotherapy. J. Control. Release Off. J. Control. Release Soc..

[6] Narayanan J.S.S., Ray P., Hayashi T., Whisenant T.C., Vicente D., Carson D.A., Miller A.M., Schoenberger S.P., White R.R. (2019). Irreversible Electroporation Combined with Checkpoint Blockade and TLR7 Stimulation Induces Antitumor Immunity in a Murine Pancreatic Cancer Model. Cancer Immunol. Res..

[7] Wang X., Yu B., Cao B., Zhou J., Deng Y., Wang Z., Jin G. (2021). A chemical conjugation of JQ-1 and a TLR7 agonist induces tumoricidal effects in a murine model of melanoma via enhanced immunomodulation. Int. J. Cancer.

[8] Wang X., Liu Y., Diao Y., Gao N., Wan Y., Zhong J., Zheng H., Wang Z., Jin G. (2018). Gastric cancer vaccines synthesized using a TLR7 agonist and their synergistic antitumor effects with 5-fluorouracil. J. Transl. Med..

[9] Beatson R.E., Taylor-Papadimitriou J., Burchell J.M. (2010). MUC1 immunotherapy. Immunotherapy.

[10] Rashidijahanabad Z., Huang X. (2020). Recent advances in tumor associated carbohydrate antigen based chimeric antigen receptor T cells and bispecific antibodies for anti-cancer immunotherapy. Semin. Immunol..

[11] Stergiou N., Urschbach M., Gabba A., Schmitt E., Kunz H., Besenius P. (2021). The Development of Vaccines from Synthetic Tumor-Associated Mucin Glycopeptides and their Glycosylation-Dependent Immune Response. Chem. Rec..

[12] Zhou S.H., Li Y.T., Zhang R.Y., Liu Y.L., You Z.W., Bian M.M., Wen Y., Wang J., Du J.J., Guo J. (2022). Alum Adjuvant and Built-in TLR7 Agonist Synergistically Enhance Anti-MUC1 Immune Responses for Cancer Vaccine. Front. Immunol..

[13] Lakshminarayanan V., Thompson P., Wolfert M.A., Buskas T., Bradley J.M., Pathangey L.B., Madsen C.S., Cohen P.A., Gendler S.J., Boons G.J. (2012). Immune recognition of tumor-associated mucin MUC1 is achieved by a fully synthetic aberrantly glycosylated MUC1 tripartite vaccine. Proc. Natl. Acad. Sci. USA.

[14] Liu Y., Tang L., Gao N., Diao Y., Zhong J., Deng Y., Wang Z., Jin G., Wang X. (2020). Synthetic MUC1 breast cancer vaccine containing a Toll-like receptor 7 agonist exerts antitumor effects. Oncol. Lett..

[15] Bianchini G., Balko J.M., Mayer I.A., Sanders M.E., Gianni L. (2016). Triple-negative breast cancer: Challenges and opportunities of a heterogeneous disease. Nat. Rev. Clin. Oncol..

[16] Beckwith D.M., Cudic M. (2020). Tumor-associated O-glycans of MUC1: Carriers of the glyco-code and targets for cancer vaccine design. Semin. Immunol..

[17] Quoix E., Lena H., Losonczy G., Forget F., Chouaid C., Papai Z., Gervais R., Ottensmeier C., Szczesna A., Kazarnowicz A. (2016). TG4010 immunotherapy and first-line chemotherapy for advanced non-small-cell lung cancer (TIME): Results from the phase 2b part of a randomised, double-blind, placebo-controlled, phase 2b/3 trial. Lancet Oncol..

[18] Guckel B., Rentzsch C., Nastke M.D., Marme A., Gruber I., Stevanovic S., Kayser S., Wallwiener D. (2006). Pre-existing T-cell immunity against mucin-1 in breast cancer patients and healthy volunteers. J. Cancer Res. Clin. Oncol..

[19] Fremd C., Stefanovic S., Beckhove P., Pritsch M., Lim H., Wallwiener M., Heil J., Golatta M., Rom J., Sohn C. (2016). Mucin 1-specific B cell immune responses and their impact on overall survival in breast cancer patients. Oncoimmunology.

[20] Liu L., Wang Y., Miao L., Liu Q., Musetti S., Li J., Huang L. (2018). Combination Immunotherapy of MUC1 mRNA Nano-vaccine and CTLA-4 Blockade Effectively Inhibits Growth of Triple Negative Breast Cancer. Mol. Ther. J. Am. Soc. Gene Ther..

[21] Beesu M., Salyer A.C., Brush M.J., Trautman K.L., Hill J.K., David S.A. (2017). Identification of High-Potency Human TLR8 and Dual TLR7/TLR8 Agonists in Pyrimidine-2,4-diamines. J. Med. Chem..

[22] Zitvogel L., Galluzzi L., Kepp O., Smyth M.J., Kroemer G. (2015). Type I interferons in anticancer immunity. Nat. Rev. Immunol..

[23] Van Gool S.W., Vandenberghe P., de Boer M., Ceuppens J.L. (1996). CD80, CD86 and CD40 provide accessory signals in a multiple-step T-cell activation model. Immunol. Rev..

[24] Guy B. (2007). The perfect mix: Recent progress in adjuvant research. Nat. Rev. Microbiol..

[25] He P., Zou Y., Hu Z. (2015). Advances in aluminum hydroxide-based adjuvant research and its mechanism. Hum. Vaccines Immunother..

[26] Akilesh H.M., Buechler M.B., Duggan J.M., Hahn W.O., Matta B., Sun X., Gessay G., Whalen E., Mason M., Presnell S.R. (2019). Chronic TLR7 and TLR9 signaling drives anemia via differentiation of specialized hemophagocytes. Science.

[27] Mailliard R.B., Egawa S., Cai Q., Kalinska A., Bykovskaya S.N., Lotze M.T., Kapsenberg M.L., Storkus W.J., Kalinski P. (2002). Complementary dendritic cell-activating function of CD8+ and CD4+ T cells: Helper role of CD8+ T cells in the development of T helper type 1 responses. J. Exp. Med..

[28] Lin W.W., Karin M. (2007). A cytokine-mediated link between innate immunity, inflammation, and cancer. J. Clin. Invest..

[29] Díaz-Zaragoza M., Hernández-Ávila R., Viedma-Rodríguez R., Arenas-Aranda D., Ostoa-Saloma P. (2015). Natural and adaptive IgM antibodies in the recognition of tumor-associated antigens of breast cancer (Review). Oncol. Rep..

[30] Sato-Kaneko F., Yao S., Ahmadi A., Zhang S.S., Hosoya T., Kaneda M.M., Varner J.A., Pu M., Messer K.S., Guiducci C. (2017). Combination immunotherapy with TLR agonists and checkpoint inhibitors suppresses head and neck cancer. JCI Insight.

[31] Stephens A.J., Burgess-Brown N.A., Jiang S. (2021). Beyond Just Peptide Antigens: The Complex World of Peptide-Based Cancer Vaccines. Front. Immunol..

