# Supplementary material for: Breast Cancer Vaccine Containing a Novel Toll-like Receptor 7 Agonist and an Aluminum Adjuvant Exerts Antitumor Effects
Source: Int J Mol Sci. 2022 Dec 1;23(23):15130. doi: 10.3390/ijms232315130 (PMC9741412; doi:10.3390/ijms232315130)
Supplement: Supplementary file 1 [file ijms-23-15130-s001.zip › ijms-1930660-supplementary.pdf]

A

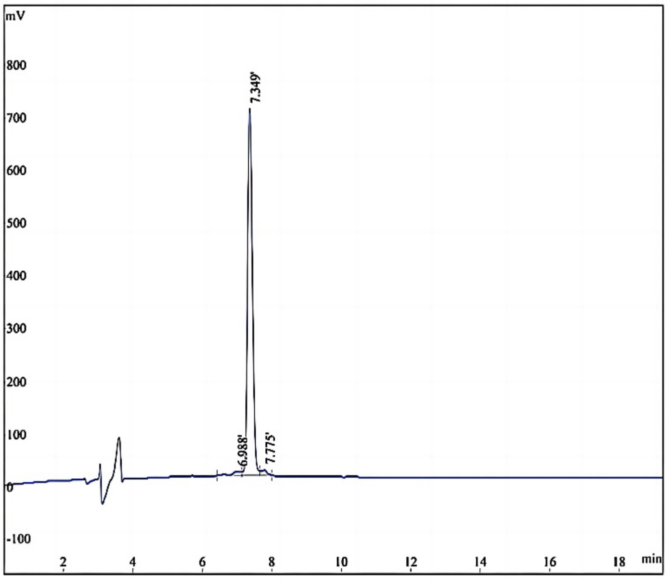

B

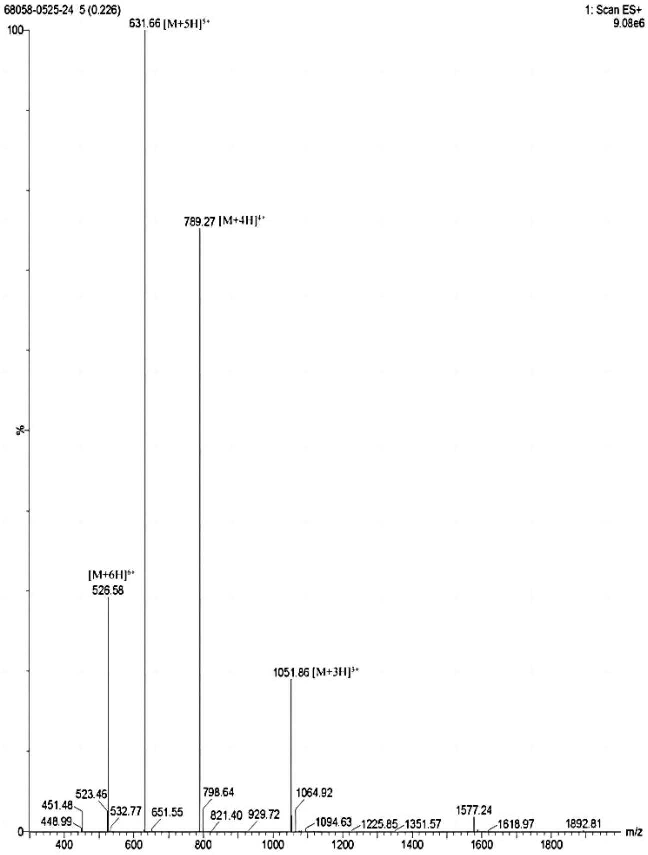

Figure S1. Certificates of analysis of MUC1 peptide. (A) Purity: 96.28%. RP-HPLC conditions: Column: Kromasil 100-5C18, 4.6 mm X 250 mm, 5 μm. Buffer A：0.1% TFA in acetonitrile; Buffer B: 0.1% TFA in water. (B) Molecular weight determined by ESI-MS, calculated 3151.72, found 1051.86 $[M+3H]^{3+}$.

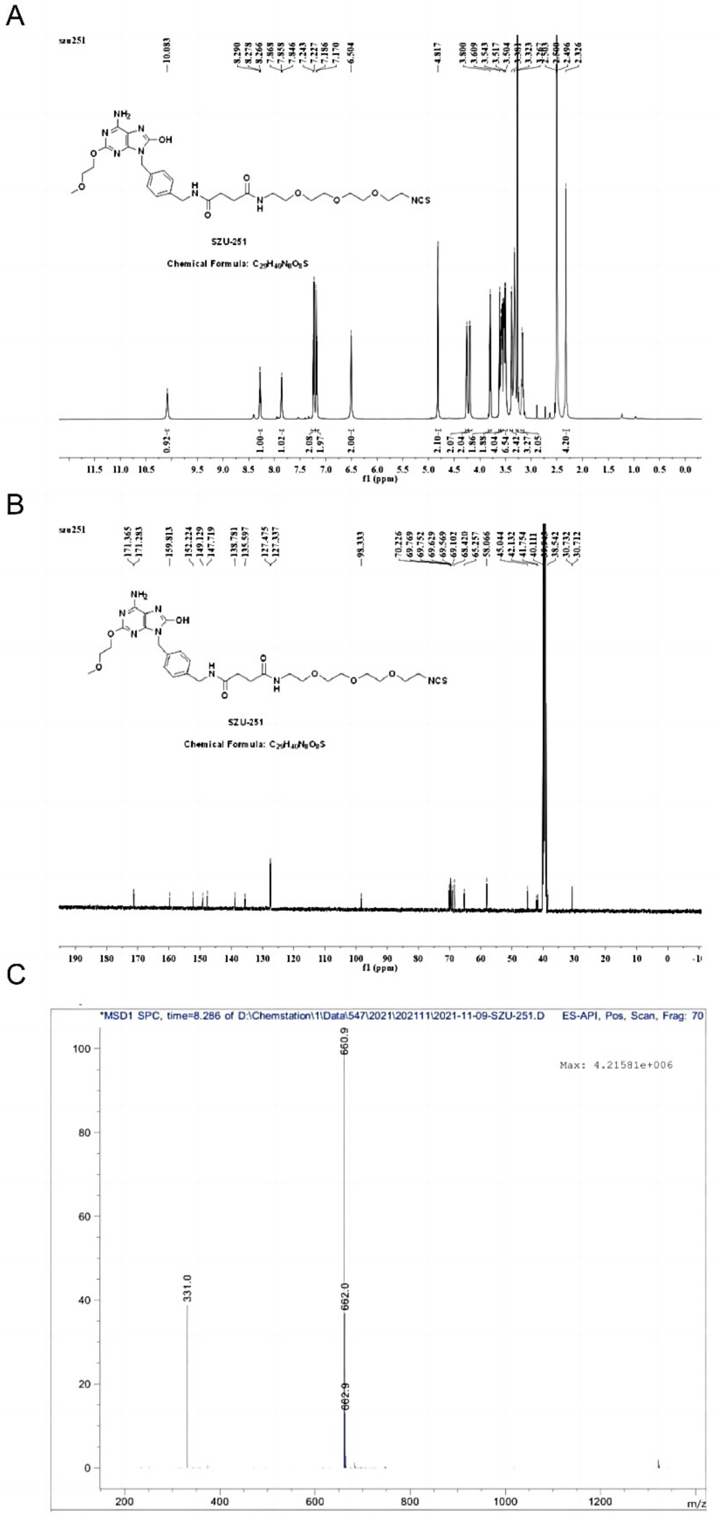

Figure S2. Structure confirmation of SZU251. (A) ^{1}H NMR. (B) ^{13}C NMR. (C) Molecular weight determined by ESI-MS, calculated 660.27, found 660.90 $[M+H]^{+}$.

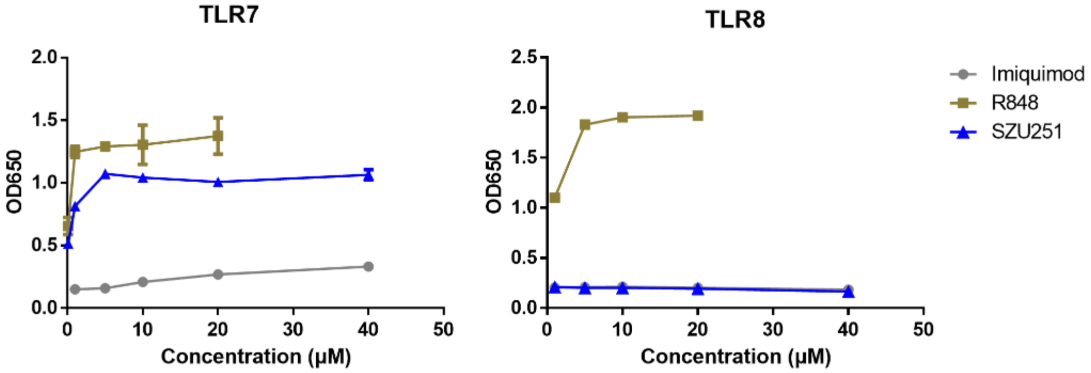

Figure S3. SZU251 stimulated TLR7 signaling. HEK-Blue hTLR7 cells and HEK-Blue hTLR8 cells were treated overnight at the indicated concentrations of the compounds, and the final OD values were recorded at 650 nm. R848 and imiquimod were used as positive controls. Data are presented as the mean ± SE; n=3.

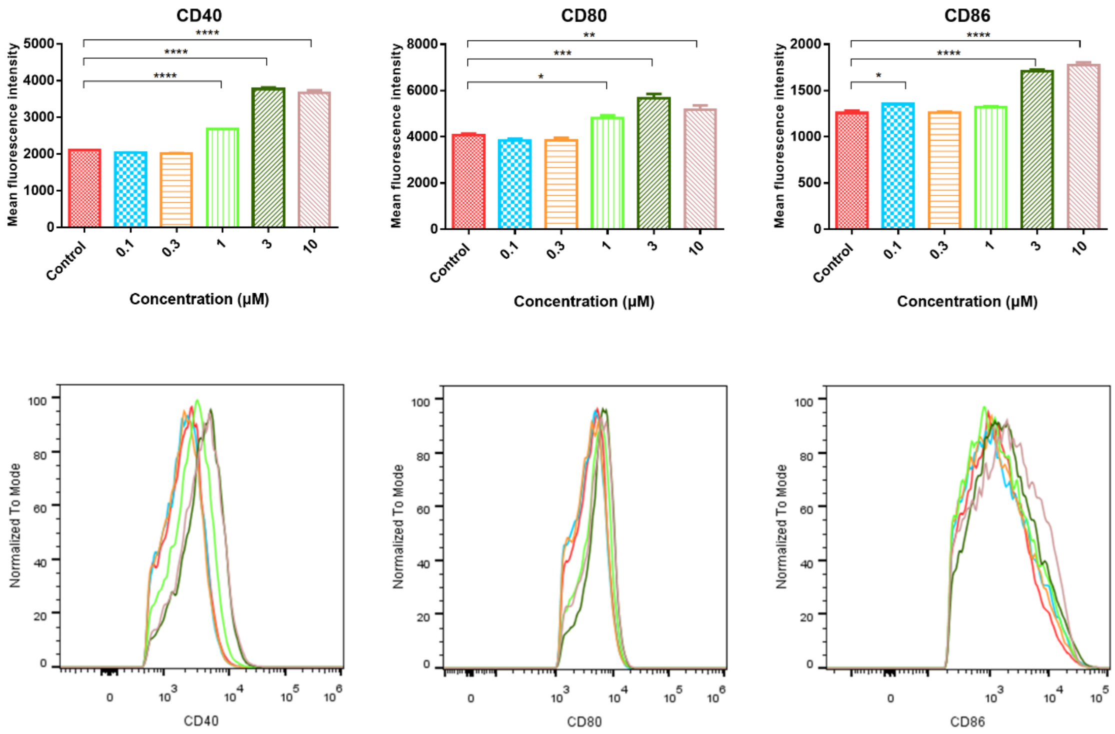

Figure S4. SZU251+MUC1 induced a concentration-dependent maturation of BMDCs, where the expression of the surface molecules, CD40, CD80 and CD86, was evaluated by flow cytometry. Data are presented as the mean ± SE; n=3. * $p < 0.05$, ** $p < 0.01$, *** $p < 0.001$, **** $p < 0.0001$.

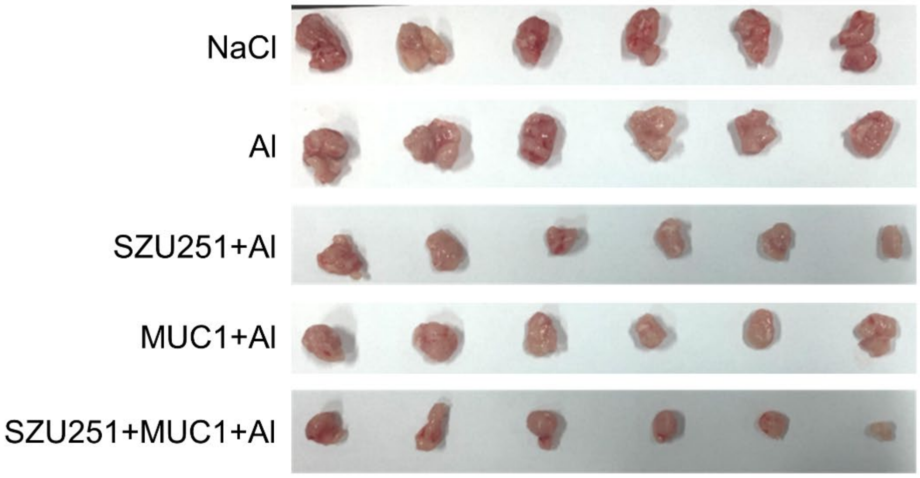

Figure S5. Representative images of the excised tumors in the prophylactic schedule.

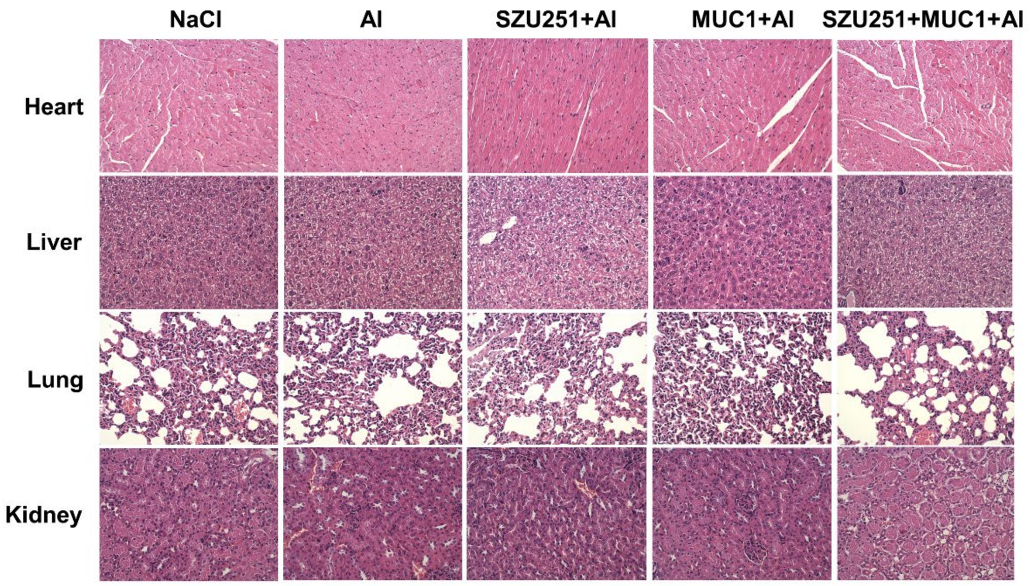

Figure S6. HE staining of the major organs of the mice in the prophylactic schedule, including hearts, livers, lungs and kidneys.

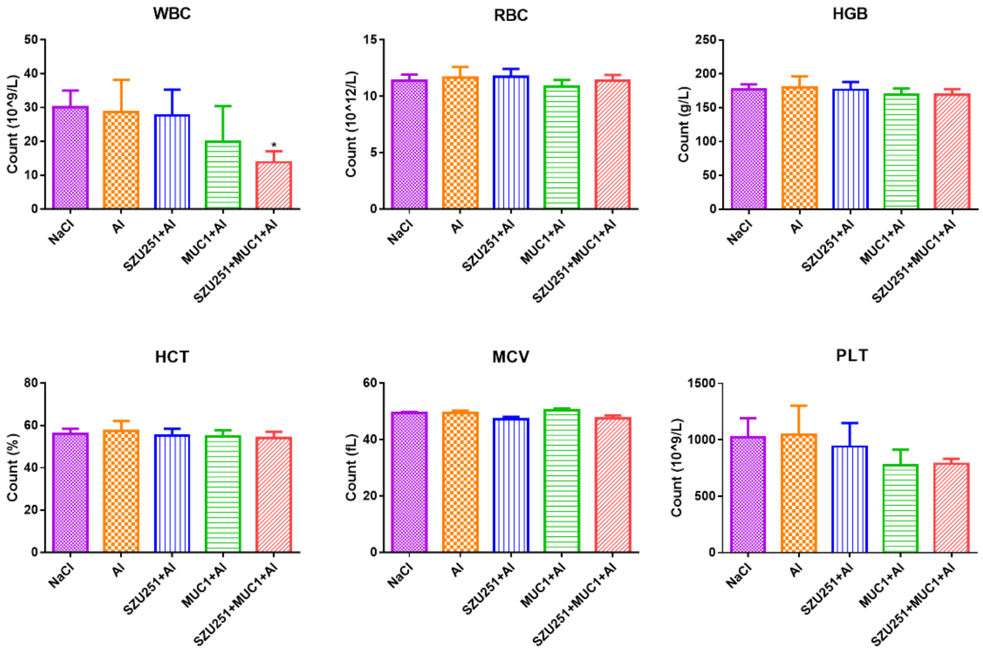

Figure S7. Blood routine examination of the mice in the prophylactic schedule, where WBC, RBC, HGB, HCT, MCV and PLT were displayed. Data are presented as the mean ± SE; n≥5.

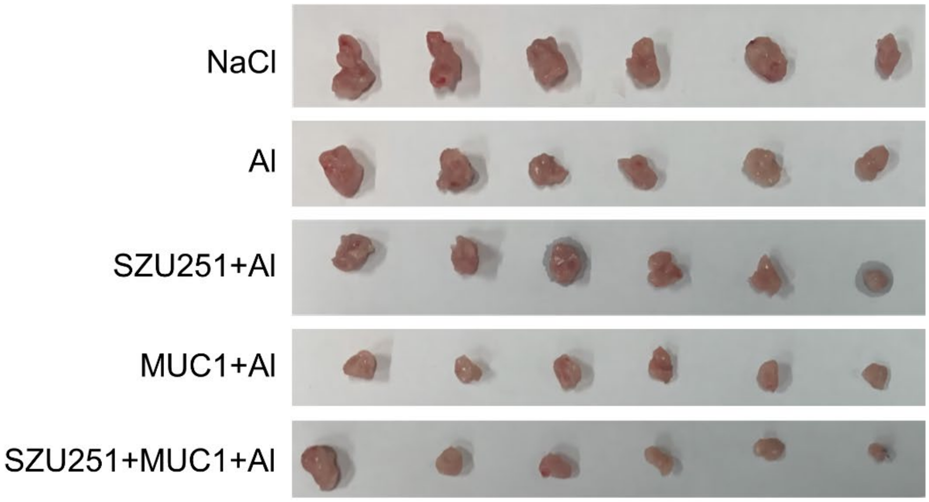

Figure S8. Representative images of the excised tumors in the therapeutic schedule.

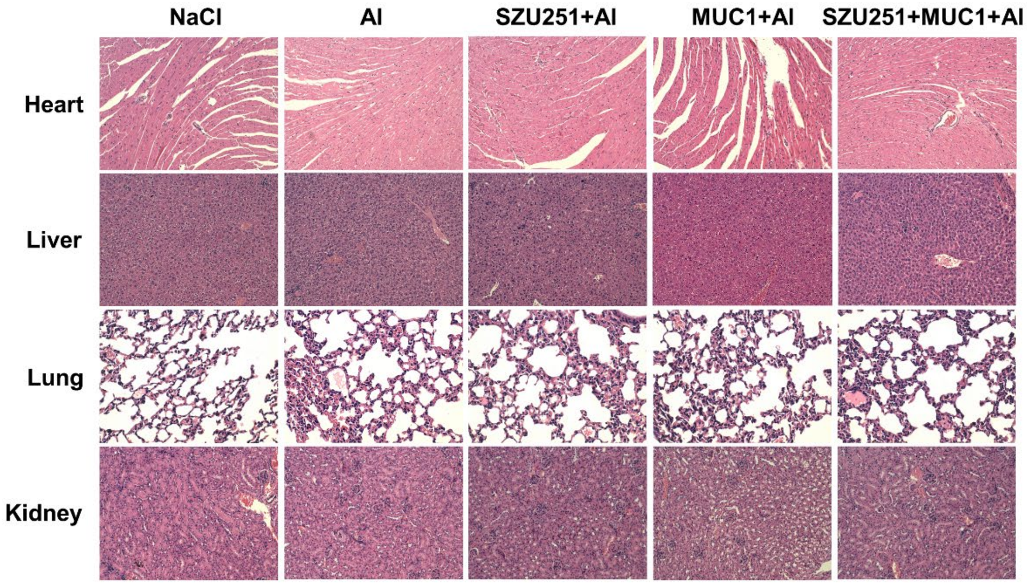

Figure S9. HE staining of the major organs of the mice in the therapeutic schedule, including hearts, livers, lungs and kidneys.

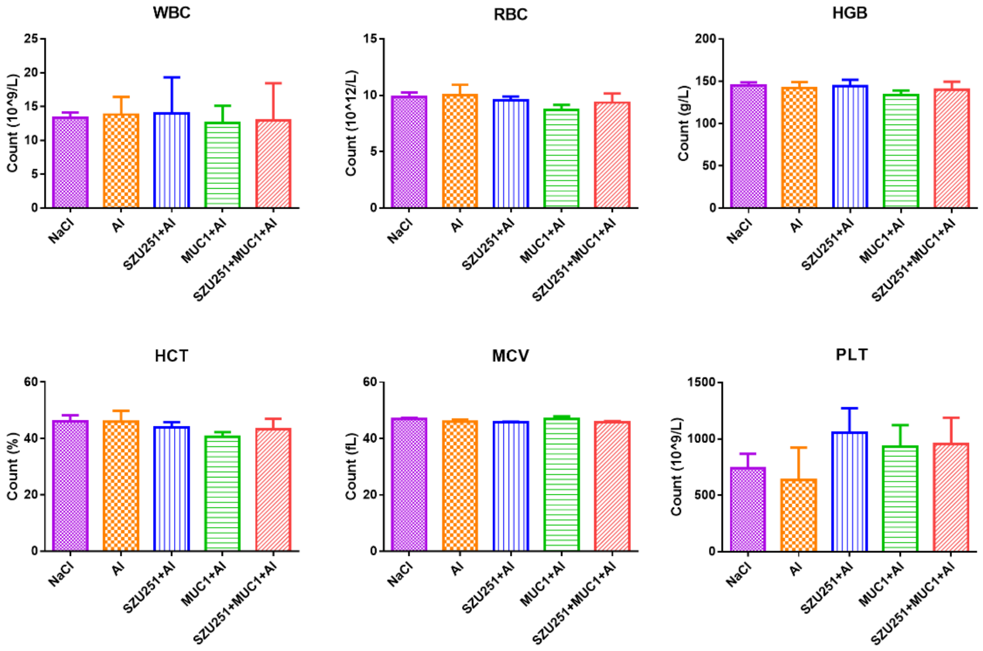

Figure S10. Blood routine examination of the mice in the therapeutic schedule, where WBC, RBC, HGB, HCT, MCV and PLT were displayed. Data are presented as the mean ± SE; n≥5.
